# Biphasic roles of pentraxin 3 in cerebrovascular function after white matter stroke

**DOI:** 10.1111/cns.13510

**Published:** 2020-12-11

**Authors:** Akihiro Shindo, Hajime Takase, Gen Hamanaka, Kelly K. Chung, Emiri T. Mandeville, Naohiro Egawa, Takakuni Maki, Mia Borlongan, Ryosuke Takahashi, Josephine Lok, Hidekazu Tomimoto, Eng H. Lo, Ken Arai

**Affiliations:** ^1^ Neuroprotection Research Laboratory Departments of Radiology and Neurology Massachusetts General Hospital and Harvard Medical School Charlestown MA USA; ^2^ Department of Neurology Mie University Graduate School of Medicine Tsu Japan; ^3^ Department of Neurology Kyoto University Graduate School of Medicine Kyoto Japan; ^4^ Pediatric Critical Care Medicine Massachusetts General Hospital Boston MA USA

**Keywords:** angiogenesis, blood‐brain barrier, neurovascular unit, pentraxin 3, stroke, white matter

## Abstract

Recent clinical studies suggest that pentraxin 3 (PTX3), which is known as an acute‐phase protein that is produced rapidly at local sites of inflammation, may be a new biomarker of disease risk for central nervous system disorders, including stroke. However, the effects of PTX3 on cerebrovascular function in the neurovascular unit (NVU) after stroke are mostly unknown, and the basic research regarding the roles of PTX3 in NVU function is still limited. In this reverse translational study, we prepared mouse models of white matter stroke by vasoconstrictor (ET‐1 or L‐Nio) injection into the corpus callosum region to examine the roles of PTX3 in the pathology of cerebral white matter stroke. PTX3 expression was upregulated in GFAP‐positive astrocytes around the affected region in white matter for at least 21 days after vasoconstrictor injection. When PTX3 expression was reduced by PTX3 siRNA, blood‐brain barrier (BBB) damage at day 3 after white matter stroke was exacerbated. In contrast, when PTX3 siRNA was administered at day 7 after white matter stroke, compensatory angiogenesis at day 21 was promoted. In vitro cell culture experiments confirmed the inhibitory effect of PTX3 in angiogenesis, that is, recombinant PTX3 suppressed the tube formation of cultured endothelial cells in a Matrigel‐based in vitro angiogenesis assay. Taken together, our findings may support a novel concept that astrocyte‐derived PTX3 plays biphasic roles in cerebrovascular function after white matter stroke; additionally, it may also provide a proof‐of‐concept that PTX3 could be a therapeutic target for white matter‐related diseases, including stroke.

## 1 INTRODUCTION

The concept of the neurovascular unit (NVU) highlights the importance of interactions between different types of cells in maintaining brain homeostasis.^1^, ^2^ Several mediators are proposed to participate in these cell‐cell interactions under both physiological and pathological conditions.^3^, ^4^, ^5^, ^6^, ^7^ After brain injury, such as stroke, many neurovascular mediators are activated, of which some exhibit biphasic properties after injury.^8^, ^9^, ^10^, ^11^ For example, VEGF and MMP‐9 cause blood‐brain barrier (BBB) damage in the acute phase of stroke and exacerbate neurovascular dysfunction, but they also promote compensatory angiogenesis in the chronic phase.^12^, ^13^, ^14^, ^15^, ^16^, ^17^, ^18^ Therefore, understanding the pathways by which neurovascular mediators exert biphasic properties after stroke is a necessary piece of solving the puzzle of stroke pathology. However, it is still unknown what and how NVU mediators exhibit biphasic effects after stroke in cerebral white matter, which is vulnerable to ischemic stress.

Recently, we proposed that pentraxin 3 (PTX3) may be an important NVU mediator that regulates stroke pathology in gray matter.^19^, ^20^ PTX3, a member of the long pentraxin family, is an acute‐phase protein (APP), which acutely increases in response to stress or inflammation. Pentraxins are defined by a pentraxin domain of 200 amino acids at their C‐terminal end. The PTX3 protein is composed of 381 amino acids (~40 kDa) and is rapidly secreted upon inflammation as a soluble pattern recognition receptor.^21^ Recent clinical studies suggest that PTX3 may be a biomarker for cardiovascular or cerebrovascular diseases^21^, ^22^, ^23^, ^24^, ^25^; however, the roles of PTX3 in the brain, especially in cerebral white matter, are mostly unknown and understudied. Because white matter damage is a clinically important aspect of cerebrovascular diseases,^26^, ^27^, ^28^, ^29^, ^30^, ^31^, ^32^, ^33^ we used mouse models of white matter stroke in a reverse translational study to examine the roles of PTX3 in stroke pathology, focusing on cerebrovascular function.

## 2 MATERIALS AND METHODS

All in vivo and in vitro experiments were performed following protocols institutionally approved by the Massachusetts General Hospital Subcommittee on Research Animal Care and in accordance with the National Institutes of Health Guide for the Care and Use of Laboratory Animals. All procedures of post postmortem brain tissue followed the Clinical Study Guidelines of the Ethics Committee of Mie University Hospital and Kyoto University Hospital and were approved by the internal review board.

### 2.1 Chemicals and reagents

Mouse recombinant PTX3 was purchased from R&D Systems. Endothelin‐1 (ET‐1), 70 kDa FITC dextran, TRITC albumin, BrdU, and mouse recombinant VEGF were purchased from Sigma. N5‐(1‐iminoethyl)‐L‐ornithine (L‐Nio), control siRNA, and mouse PTX3 siRNA were purchased from Santa Cruz. PepJet DNA in vivo transfection reagent was purchased from SignaGen Laboratories. Lipofectamine 2000 transfection reagent was purchased from Thermo Fisher Scientific.

### 2.2 Postmortem human brain sections

Ten autopsied brains (five patients with ischemic stroke [three males, two females, and ages 80.0 ± 6.7] and five patients with nonstroke pathologies [amyotrophic lateral sclerosis patients; two males, three females, and ages 75.6 ± 8.8]) were obtained from Mie and Kyoto University Hospital. Neuropathological diagnoses were made by histopathological examination. In patients with cerebral infarction, death occurred 4‐8 weeks after stroke onset, and PTX3 expression in the peri‐infarct regions of subcortical white matter was evaluated in the postmortem brains. As a control, the same region of subcortical white matter in postmortem brains of patients with amyotrophic lateral sclerosis (no ischemic damage) was assessed. For immunostaining, formalin‐fixed 6‐μm‐thick brain sections were prepared. After blocking with 3% BSA, these brain sections were incubated in phosphate‐buffered saline (PBS)/0.1% Tween solution containing primary antibodies (anti‐PTX3 [1:100, Enzo] and anti‐GFAP [1:100, BD Pharm]) at 4°C overnight. Then, the sections were washed three times with PBS and incubated with secondary antibodies with fluorescence conjugations at room temperature for 1 hour. Immunoreactive signals were detected with a fluorescence microscope interfaced with a digital charge‐coupled device camera. The staining areas of PTX3 were analyzed by quantifying the mean intensity area for five partitions of each brain section, using ImageJ analysis software by an operator who was blinded to the group allocation.

### 2.3 Mouse models of white matter stroke

Male C57BL/6 mice (8‐9 weeks old, weighing 22‐26 g, Charles River Laboratories) were anesthetized by isoflurane in a 70% N_2_O and 30% O_2_ mixture. Mice with white matter stroke were prepared as described previously^34^, ^35^ with some modifications. Briefly, 1 μL of ET‐1 solution (10 μg/mL) or L‐Nio solution (27 mg/mL) was injected through a 0.2‐mm needle over 5 minutes into the right corpus callosum (anterior: 0.5 mm from bregma, lateral: 1.0 mm from bregma, depth: 2.1 mm from skull surface). Mice in the sham group received the same volume of PBS. For the loss‐of‐function experiments, control siRNA or PTX3 siRNA in the PepJet DNA transfection reagent was injected into the right intracerebroventricular (ICV) over 1 minute (posterior: 0.5 mm from bregma, lateral 1.0 mm from bregma, depth: 2.0 mm from the skull surface). To detect newly generated CD31‐positive endothelial cells, BrdU (50 mg/kg) was injected intraperitoneally twice a day between 6 and 14 days after white matter stroke, according to our previous study.^36^ All in vivo experiments were performed in a blinded manner. The number of animals used for each experiment is stated in the figure legends.

### 2.4 Evaluation of BBB leakage by FITC dextran and TRITC albumin

Three days after white matter stroke, 1% 70 kDa FITC dextran and 1% TRITC albumin were administered intravenously to the animals. Two hours later, the brains were quickly removed and snap‐frozen. Coronal sections of 16 μm thickness were prepared using a cryostat (Microm HM505E) at −20°C and stored at −80°C until use. Fluorescent signals of these sections were analyzed by a fluorescence microscope interfaced with a digital charge‐coupled device camera. To measure the area of fluorescent staining, one coronal brain section from the region + 0.4~+0.6 µm to the bregma was randomly selected per animal, and one 500 µm square ROI was defined, wherein the ET‐1 or L‐Nio injection point was set as the center. The areas with FITC dextran and TRITC albumin were analyzed by an operator who was blinded to the group allocation, using ImageJ analysis software.

### 2.5 Immunohistochemistry, IgG, and Fluoromyelin staining

Brain sections from mice with white matter stroke were fixed by 4% paraformaldehyde and rinsed with PBS. After blocking with 3% BSA, sections were incubated at 4°C overnight in PBS/0.1% Tween solution containing primary antibodies: anti‐PTX3 (1:100, Enzo), anti‐GFAP (astrocyte marker, 1:100, BD Pharm), anti‐NeuN (neuron marker, 1:100, Chemicon), anti‐PDGFRα (OPC marker, 1:100, Santa Cruz), anti‐CC1 (oligodendrocyte marker, 1:100, Calbiochem), anti‐PDGFRβ (pericyte marker, 1:100, R&D Systems), anti‐CD31 (endothelial cell marker, 1:100 BD Pharm), anti‐ZO‐1 (1:100, Invitrogen), anti‐BrdU (1:100, Invitrogen, 1:100, AbD Serotec), anti‐collagen IV(1:100, Southern Biotech), or anti‐VEGFR2 (1:100, Abcam). For IgG staining, sections were incubated overnight at 4°C with antibody against donkey anti‐rat IgG (1:300, Jackson Immunoresearch Laboratories). Then, sections were washed with PBS and incubated with secondary antibodies with fluorescence conjugations at room temperature for 1 hour. For myelin staining, sections were incubated with FluoroMyelin Red fluorescent myelin stain (1:300, Thermo Fisher) for 20 minutes at room temperature and washed out thereafter. Immunostaining was analyzed with fluorescence microscope interfaced with a digital charge‐coupled device camera. To measure the intensity of fluorescent staining, one coronal brain section from the region + 0.4~+0.6 µm to the bregma was randomly selected per animal, and one 500 µm square ROI was defined, wherein the ET‐1 or L‐Nio injection point was set as the center. The images were analyzed by an operator who was blinded to the group allocation, using ImageJ analysis software.

### 2.6 Western blot

Corpus callosum samples from white matter stroke mice were homogenized in Pro‐PREP^TM^ Protein Extraction solution (Boca scientific). Western blot samples were mixed with equal volumes of SDS (Novex) sample buffer containing 10% 2‐mercaptoethanol (Sigma). Each sample was loaded into 4%‐20% Tris‐glycine gels (Novex). After electrophoresis and transferring to nitrocellulose membranes (Novex), the membranes were blocked in I‐block (Tropix) for 60 minutes and then incubated overnight at 4°C with primary antibodies with PTX3 (1:1000) or β‐actin (1:5000, Sigma Aldrich), followed by incubation with peroxidase‐conjugated secondary antibodies. Signals were visualized with chemiluminescence (GE Healthcare), and the optical density of Western blot bands was assessed by an operator who was blinded to the group allocation, using ImageJ analysis software.

### 2.7 Cell culture

Rat brain microendothelial cell line RBE.4 cells were prepared according to our previous report.^19^ Briefly, RBE.4 cells were maintained in endothelial basal medium‐2 (EBM‐2, Lonza) supplemented with EGM‐2MV SingleQuots kit (Lonza) and seeded on collagen‐coated plates and incubated in a 5% CO_2_ incubator at 37°C. These RBE.4 cells were used for Matrigel tube formation assay.

### 2.8 Matrigel tube formation assay

The standard Matrigel method was used as a surrogate in an in vitro angiogenesis assay to assess the spontaneous formation of capillary like structures of RBE.4 cells. Standard 15‐well plates were coated with 12 μL of cold Matrigel and allowed to be solidified at 37°C for 30 minutes. Outgrowth RBE.4 cells (2 × 10^5^ cells/mL) were seeded into the plates and incubated at 37°C for 18 hours with concentrations of rPTX3 (0 to 10 ng/mL), rVEGF (10 ng/mL), and rPTX3 with rVEGF (both 10 ng/mL). The degree of tube formation was determined by counting the number of tubes in five random fields from each well under 10x magnification, as described previously.^37^ The number of tubes was counted by an operator who was blinded to the group allocation.

### 2.9 Statistical analysis

Results were expressed as mean ± SD The Shapiro‐Wilk test was used to check the distribution of our data. To compare between two groups, statistical significance was evaluated using Unpaired *t* test with Welch's correction for groups of normal distribution or Mann‐Whitney *U* test for groups of non‐normal distribution. For groups of normal distribution, multiple group comparisons of continuous variables were performed by one‐way analysis of variance followed by post hoc Dunnett (for time‐course analyses) or Tukey (for the others) tests; for groups of non‐normal distribution, Kruskal‐Wallis test was performed followed by post hoc Steel‐Dwass test. A *P* value of <.05 was considered statistically significant.

## 3 RESULTS

**FIGURE 1:**
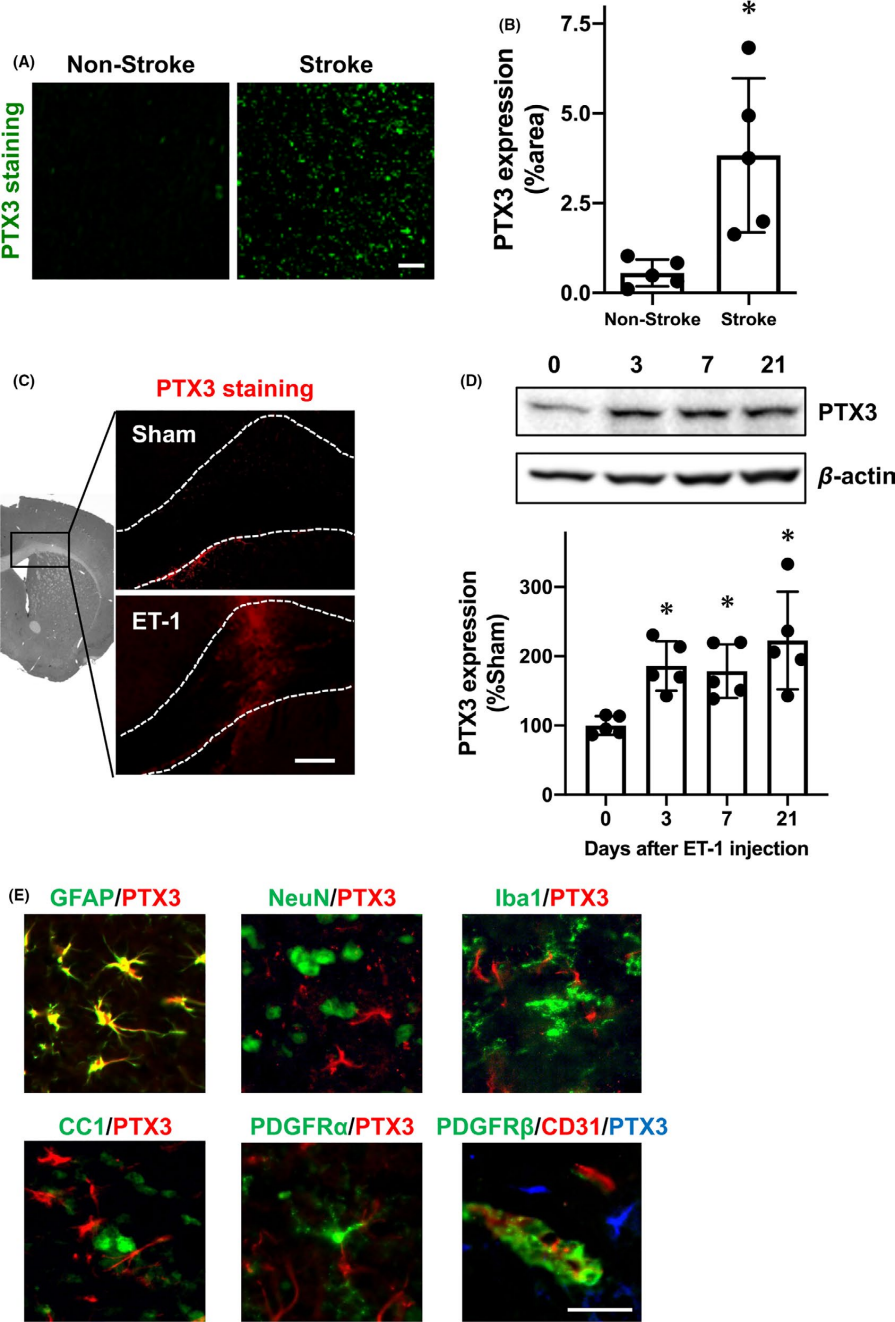
Pentraxin 3 expression after white matter stroke: A‐B, Postmortem brain tissue with ischemic stroke showed upregulation of PTX3 in the peri‐infarct area of white matter, whereas tissues without stroke expressed infrequent PTX3 in subcortical white matter. Scale bar = 100 μm. Data are mean ± SD from N = 5. *P < .05 (unpaired *t* test with Welch's correction). A, C, Immunostaining showed that PTX3 expression was increased at day 3 after white matter stroke caused by ET‐1 injection in mice. Scale bar = 100 μm. D, Western blots showed that PTX3 increased after ET‐1 injection was sustained for up to at least 21 d. Data are mean ± SD from N = 5. *P < .05 vs sham (one‐way ANOVA followed by post hoc Dunnett's multiple comparison test). E, Double staining of PTX3 with cell‐specific marker antibodies showed that the major cell type of PTX3 expression at day 3 after ET‐1 injection was GFAP‐positive astrocytes. Scale bar = 50 μm

We first examined PTX3 expression levels in cerebral white matter after stroke in human and mouse samples. Immunostaining using postmortem brain tissues showed that larger numbers of PTX3 positive cells were detected in the white matter region in stroke patients compared to nonstroke patients (Figure 1A‐B). Similarly, in a mouse model of white matter stroke (ET‐1 injection into corpus callosum), PTX3 expression was increased in the corpus callosum at day 3 after stroke (Figure 1C). Western blotting using corpus callosum samples from stroke mice showed that the increased expression of PTX3 was sustained until at least day 21 after injury (Figure 1D). In addition, double staining of PTX3 with cell‐specific markers (GFAP for astrocyte, NeuN for neuron, Iba‐1 for microglia/macrophage, CC‐1 for oligodendrocyte, PDGFR‐α for oligodendrocyte precursor cell (OPC), PDGFR‐β for pericyte, and CD31 for endothelial cell) confirmed that the majority of PTX3‐positive cells were co‐immunostained with GFAP‐positive astrocytes (Figure 1E), suggesting that astrocytes comprise the majority of PTX3‐positive cells.

**FIGURE 2:**
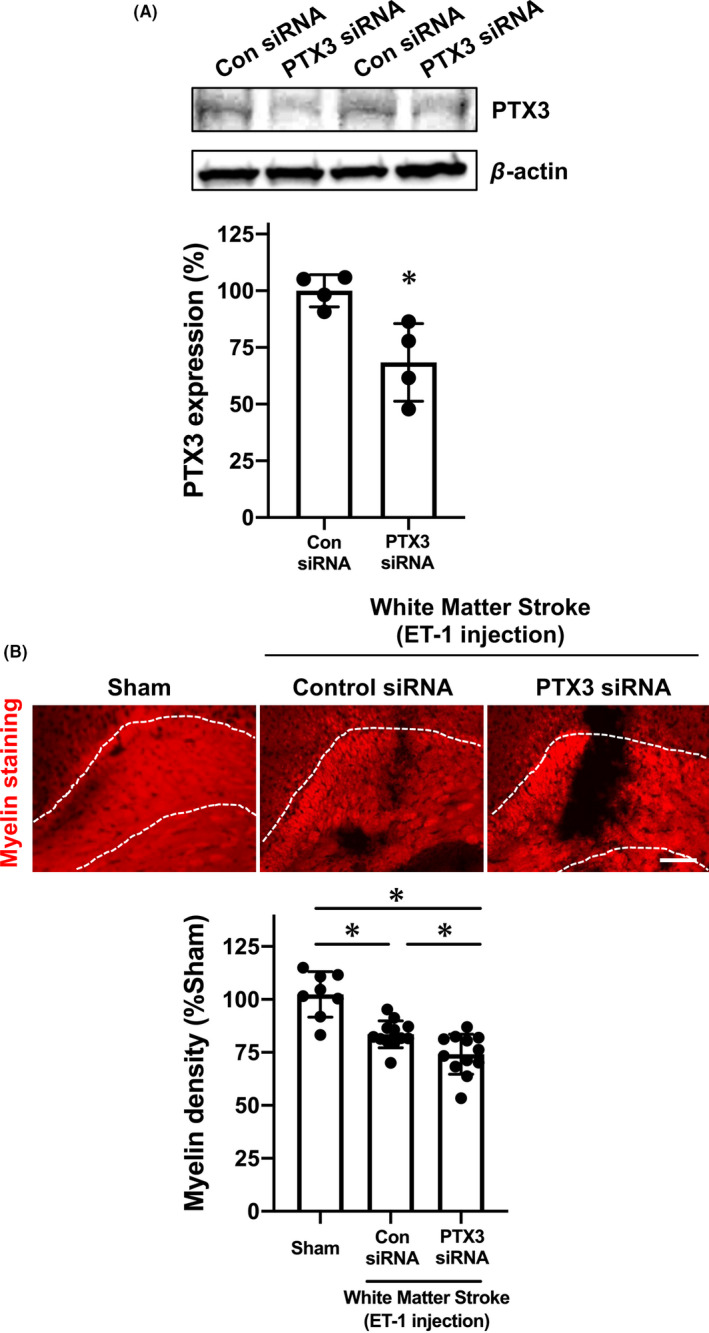
Pentraxin 3 and myelin density during the acute phase of white matter stroke in mice: Male mice were subjected to ET‐1 injection after the treatment of control siRNA or PTX3 siRNA. Three days after ET‐1 injection, white matter stroke mice were sacrificed, and brain samples were used for Western blotting or immunostaining. A, Western blotting confirmed that PTX3 siRNA downregulated PTX3 expression in the affected region at day 3 after ET‐1 injection. Data are mean ± SD from N = 4. *P < .05 (unpaired t test with Welch's correction). B, Myelin staining showed that ET‐1 injection caused myelin damage in the corpus callosum at day 3 after ET‐1 injection, and mice that received PTX3 siRNA exhibited less myelin density compared to mice with control siRNA. Scale bar = 100 μm. Data are mean ± SD from N = 8 for sham and N = 12 for ET‐1/control siRNA and ET‐1/PTX3‐siRNA. *P < .05 (one‐way ANOVA followed by post hoc Tukey's multiple comparison test)

**FIGURE 3:**
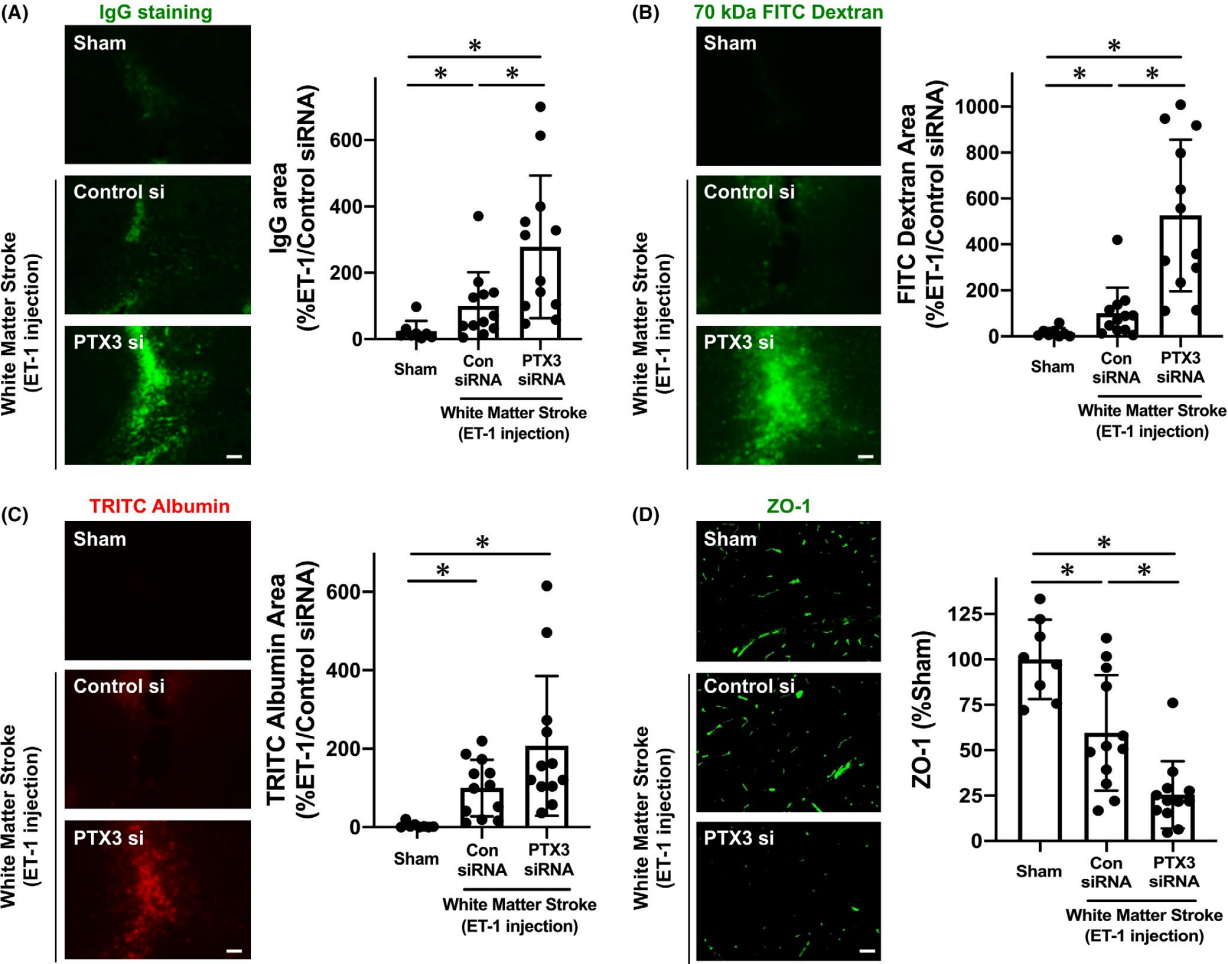
Pentraxin and BBB damage during the acute phase of white matter stroke in mice: Male mice were subjected to ET‐1 injection after the treatment of control siRNA or PTX3 siRNA. Three days after ET‐1 injection, white matter stroke mice were sacrificed, and brain samples were used for immunostaining. A, IgG staining showed that ET‐1 injection caused BBB damage in the corpus callosum at day 3 after ET‐1 injection, and mice that received PTX3 siRNA exhibited a larger extent of BBB damage compared to mice with control siRNA. Scale bar = 50 μm. Data are mean ± SD from N = 8 for sham and N = 12 for ET‐1/control siRNA and ET‐1/PTX3‐siRNA. *P < .05 (Kruskal‐Wallis followed by post hoc Steel‐Dwass test). B, Mice that received PTX3 siRNA showed more leakage of 70 kDa FITC dextran at day 3. Scale bar = 50 μm. Data are mean ± SD from N = 8 for sham and N = 12 for ET‐1/control siRNA and ET‐1/PTX3‐siRNA. *P < .05 (Kruskal‐Wallis followed by post hoc Steel‐Dwass test). C, Similarly, mice that received PTX3 siRNA also showed more leakage of TRITC albumin at day 3. Scale bar = 50 μm. Data are mean ± SD from N = 8 for sham and N = 12 for ET‐1/control siRNA and ET‐1/PTX3‐siRNA. *P < .05 (Kruskal‐Wallis followed by post hoc Steel‐Dwass test). D, Concomitantly, the expression level of a tight junction accessory protein ZO‐1 was lower in the mice with PTX3‐siRNA. Scale bar = 50 μm. Data are mean ± SD from N = 8 for sham and N = 12 for ET‐1/control siRNA and ET‐1/PTX3‐siRNA. Please see Figure S2 for the confirmation that ZO‐1 expression was observed in CD31‐positive cells in mouse corpus callosum. *P < .05 (Kruskal‐Wallis followed by post hoc Steel‐Dwass test)

To evaluate the roles of PTX3 in the pathology of white matter stroke, we injected PTX3 siRNA into the cerebral ventricle to downregulate the expression of PTX3. Western blotting confirmed that our siRNA approach successfully reduced the levels of PTX3 expression in the corpus callosum under normal conditions (Figure S1). Additionally, our PTX3 siRNA approach worked in the mouse model of white matter stroke (Figure 2A), and importantly, mice that received PTX3 siRNA on day 0 showed exacerbated myelin damage/loss on day 3 after stroke compared to mice with control siRNA injection (Figure 2B). We then examined BBB tightness by assessing IgG leakage, and we showed that the BBB was loosened in the corpus callosum on day 3 after ET‐1 injection (Figure 3A). Notably, mice with the PTX3 siRNA injection exhibited more IgG leakage (Figure 3A), suggesting that endogenous PTX3 may support BBB tightness in the acute phase of stroke. Furthermore, when 70 kDa FITC dextran or TRITC albumin was intravenously injected 2 hours before sacrifice of mice on day 3, the leakage of both molecules into brain parenchyma was increased by PTX3 downregulation (Figure 3B‐C). This loss of BBB tightness associated with PTX3 downregulation was due at least in part to the dysregulation of tight junction accessory proteins, suggested by a decrease in ZO‐1 expression after stroke in mice that received PTX3 siRNA (Figure 3D).

**FIGURE 4:**
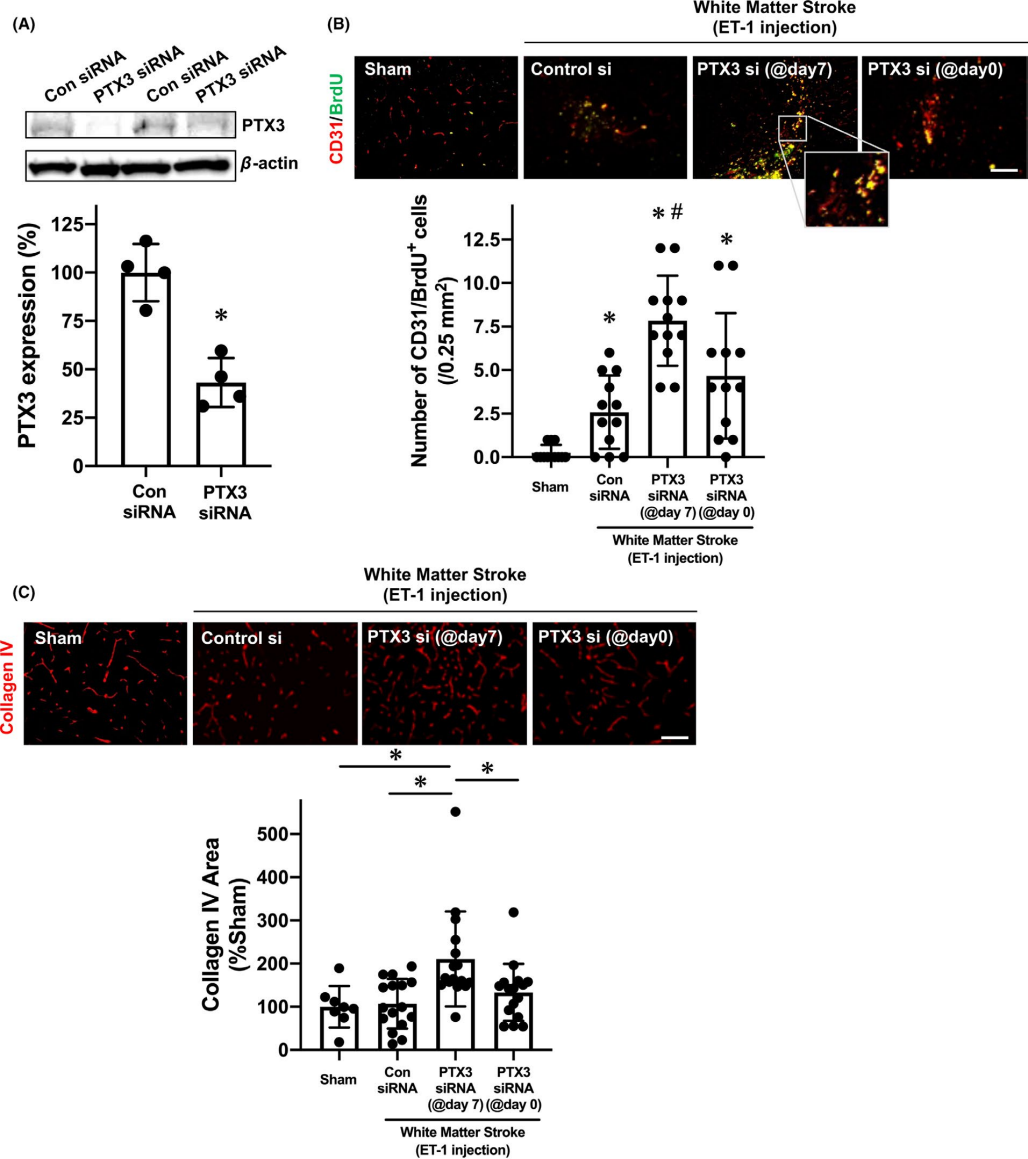
Pentraxin and compensatory angiogenesis in chronic phase of white matter stroke in mice: Male mice were subjected to ET‐1 injection, and 7 d later, these mice were treated with either control siRNA or PTX3 siRNA. As a negative control, mice were subjected to ET‐1 injection after the treatment of PTX3 siRNA. Twenty‐one days after ET‐1 injection, white matter stroke mice were sacrificed, and brain samples were used for Western blotting or immunostaining. Please see Figure S2 for the experimental diagram of this experiment. A, Western blotting confirmed that when PTX3 siRNA was treated at day 7 after ET‐1 injection, PTX3 expression in the affected region was downregulated at day 10 after ET‐1 injection. Data are mean ± SD from N = 4. *P < .05 (unpaired *t* test with Welch's correction). B, CD31/BrdU‐double staining showed that mice with PTX3‐siRNA (@day 7) had a larger number of CD31/BrdU‐double positive cells (newly generated endothelial cells) at day 21 after white matter stroke, compared to mice with control siRNA (@day 7) or mice with PTX3‐siRNA (@day 0). The inset is an enlarged CD31/BrdU‐double staining image, which confirmed that some of BrdU‐positive cells were merged with CD31 staining. Scale bar = 100 μm. Data are mean ± SD from N = 12. *P < .05 vs Sham, ^#^ *P* < .05 vs ET‐1/Control siRNA (Kruskal‐Wallis followed by post hoc Steel‐Dwass test). C, Similarly, PTX3‐siRNA (@day 7) promoted compensatory angiogenesis at day 21 after ET‐1 injection. Scale bar = 100 μm. Data are mean ± SD from N = 8 for Sham and N = 16 for other groups. *P < .05 (Kruskal‐Wallis followed by post hoc Steel‐Dwass test)

Because our data implied that endogenous PTX3 may affect the cerebrovascular system in the acute phase of white matter damage, we next examined whether PTX3 also plays some roles in the cerebrovascular system in the subacute/chronic phase of white matter stroke. When PTX3 siRNA was administered at day 7 after ET‐1 injection, the PTX3 expression around the affected region was decreased at least until day 10 (Figure 4A). With this experimental system, we examined the roles of PTX3 in compensatory angiogenesis responses after white matter stroke (see Figure S3 for the experimental diagram). The number of newly emerged endothelial cells after white matter stroke was assessed by BrdU/CD31 staining. Compared to mice that received control siRNA injection at day 7, mice that received PTX3 siRNA injection at day 7 showed larger numbers of BrdU/CD31‐double positive cells (newly emerged endothelial cells) at day 21 after stroke onset (Figure 4B). On the other hand, no change was seen in the number of BrdU/CD31‐double positive cells when mice received PTX3 siRNA administration on day 0 (Figure 4B). Furthermore, mice with PTX3 siRNA injection on day 7 showed stronger signals of collagen IV (a marker for blood vessels) on day 21 after ET‐1 injection, compared to mice with control siRNA (Figure 4C). In contrast to mice that received PTX3 siRNA on day 7, mice that received PTX3 siRNA injection on day 0 did not exhibit a higher blood vessel length/area on day 21 after ET‐1 injection, compared to mice that received control siRNA (Figure 4C). These results show that a reduction of PTX3 expression during the subacute phase led to an increase in the compensatory angiogenic responses in the chronic phase. This also indicates that a similar reduction of PTX3 expression during the acute phase did not have any effect on compensatory angiogenesis, although it resulted in decreased BBB integrity. Notably, the biphasic effects of PTX3 in the cerebrovascular system after white matter stroke—beneficial for BBB tightness during the acute phase, deleterious for angiogenic responses during the subacute/chronic phase—were confirmed by experiments using another mouse model of white matter stroke prepared by L‐Nio injection into the corpus callosum (Figure S4).

**FIGURE 5:**
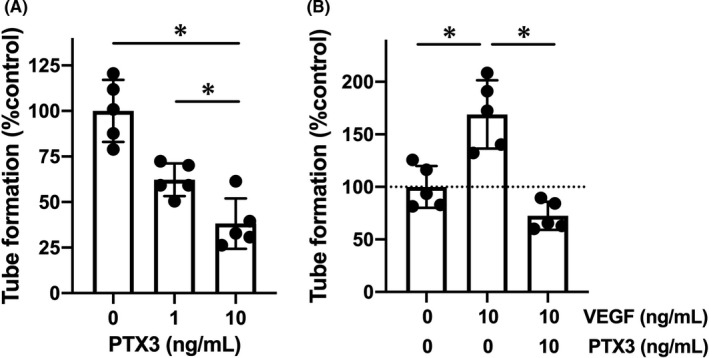
Pentraxin and in vitro angiogenesis: Brain endothelial RBE.4 cells were used for the Matrigel tube formation assay. A, RBE.4 cells were maintained in culture medium containing recombinant PTX3 for 18 h. PTX3 treatment suppressed the tube formation in a dose‐response manner. Data are mean ± SD from N = 5. *P < .05 (one‐way ANOVA followed by post hoc Tukey's multiple comparison test). B, Recombinant VEGF (10 ng/mL for 18 h) promoted the tube formation, but cotreatment with recombinant PTX3 (10 ng/mL) suppressed VEGF‐induced tube formation. Data are mean ± SD from N = 5. *P < .05 (one‐way ANOVA followed by post hoc Tukey's multiple comparison test)

Finally, we conducted cell culture experiments to verify the suppressive effects of PTX3 in angiogenesis. In the in vitro Matrigel assay system, recombinant PTX3 inhibited tube formation by cultured endothelial cells in a dose‐response manner (Figure 5A). Furthermore, recombinant PTX3 also exerted suppressive effects even under conditions of VEGF‐containing cell culture medium, that is, VEGF promoted tube formation in the Matrigel system, but cotreatment with recombinant PTX3 counteracted the pro‐angiogenic effects of VEGF (Figure 5B).

## 4 DISCUSSION

The concept of the neurovascular unit emphasizes that cell‐cell interactions between different types of cells are essential for maintaining brain homeostasis. The trophic coupling within the neurovascular unit is partly mediated by an exchange of soluble factors. Under pathological conditions, the pattern of released soluble factors within the neurovascular unit undergoes significant changes, which result in either exacerbation of brain damage or promotion of brain repair/remodeling in a context‐dependent manner. In the present study, we showed that (i) PTX3 was upregulated after white matter stroke in both human and mouse, (ii) in the acute phase of white matter stroke in mice, PTX3 supported BBB integrity, and (iii) on the other hand, PTX3 suppressed angiogenesis during the repairing phase after white matter stroke. Our findings lead to the proposal that PTX3, expressed by GFAP‐positive astrocytes, plays an important role as an NVU mediator that regulates stroke pathology through its biphasic effects after white matter stroke.

The mechanisms of stroke pathology are very complex. During the acute phase of stroke, the initiation of several deleterious cascades causes brain damage. On the other hand, during the chronic phase, some compensative responses, such as angiogenesis and neurogenesis, work to repair damaged brain tissue. Notably, some soluble factors play both harmful and beneficial roles within the neurovascular unit.^8^, ^9^, ^38^ For example, MMP‐9 is known to be released after stroke, causing NVU dysfunction by disassembling the extracellular matrix.^14^, ^39^, ^40^ However, these events caused by MMP‐9 during the acute phase of stroke appear to be necessary for compensative angiogenesis and neurogenesis during the chronic phase.^16^, ^41^ Similarly, VEGF causes BBB damage during the acute phase of stroke but promotes angiogenesis during the repair phase.^38^, ^42^ This biphasic property of NVU mediators may be a key to understanding the complicated mechanisms of stroke pathology.^15^ Our current study provides an additional example of an NVU mediator that shows biphasic properties after stroke. Another novel finding in this study is that PTX3 appears to have biphasic activities that are opposite to those of well‐known biphasic NVU mediators such as VEGF and MMP‐9. VEGF and MMP‐9 are detrimental to BBB integrity but beneficial for compensatory angiogenesis, whereas PTX3 is beneficial for BBB integrity during the acute phase of stroke but harmful for compensative angiogenic responses during the chronic phase. We and others previously showed that PTX3 can bind VEGF and other growth factors to neutralize their effects,^19^, ^43^ which suggest the existence of a complex system of counter‐regulatory effects supported by these biphasic mediators. Future studies are warranted to investigate how PTX3 interacts with NVU mediators to maintain neurovascular homeostasis.

Our reverse translational study reveals the biphasic roles of PTX3 after white matter stroke and provides a proof‐of‐concept that PTX3 can be a therapeutic target for stroke. However, there are some caveats/limitations in our study that need to be considered in future studies. First, we showed that GFAP‐positive astrocytes are a major source of PTX3 during the acute phase of white matter stroke. This finding is consistent with our previous report, which demonstrated that GFAP‐positive astrocyte was a major cell type for PTX3 expression in the cortex (eg, gray matter) after ischemic stroke in a rat model of focal ischemia.^19^ Nevertheless, it is possible that this cell specificity for PTX3 expression after brain damage may depend on the time‐course and/or disease types. Therefore, the different cell types, including pericytes and astrocytes,^6^, ^44^ that contribute to PTX3 production under diseased conditions should be tested at multiple time points in disease models other than stroke. Second, we showed that PTX3 downregulation decreased the level of ZO‐1 under conditions of white matter stroke, but the regulatory mechanism of PTX3 in the expressions of tight junction proteins remains an important question. Because PTX3 binds and inhibits the BBB‐damaging factor VEGF,^19^, ^20^, ^43^ it is possible to assume that PTX3 downregulation would accelerate the deleterious effect of VEGF in BBB tightness, resulting in the decrease of ZO‐1 expression level. However, there may be other regulatory mechanisms that influence PTX3 effects on ZO‐1 expression. Therefore, future studies are needed to examine the roles of PTX3 in tight junction proteins other than ZO‐1, including claudins and occludin, to further our understandings of PTX3 roles in BBB function. Third, our experimental system of in vivo angiogenesis assays did not identify whether the newly generated endothelial cells by PTX3 downregulation were truly “functional/effective” or not. For the purpose of drug development, whether newly generated endothelial cells (and vessels) by PTX3 siRNA contributes to the recovery of cerebral blood flow and neurological function will need to be carefully assessed in future studies.^45^, ^46^, ^47^, ^48^ In addition, although our study focuses only on angiogenetic responses to examine the roles of PTX3 in compensatory mechanisms after white matter stroke, brain recovery requires the coordinated responses of many different processes, including angiogenesis and neurogenesis.^49^, ^50^ Also, because cerebral white matter consists mainly of axonal bundles surrounded by a myelin sheath, the process of oligodendrogenesis through proliferation/differentiation of oligodendrocyte precursor cells is crucial in the development of effective therapies for white matter stroke.^33^, ^51^, ^52^ Hence, to develop an effective drug for promoting stroke recovery, these compensatory responses will need to be thoroughly assessed in preclinical studies. Finally, although our current study showed that PTX3 downregulation by PTX3 siRNA promoted compensatory angiogenesis, a previous study reported that PTX3 ko mice exhibited less angiogenesis after stroke.^53^ There may be several potential reasons for this discrepancy, but one may assume that complete knockdown before birth would change multiple signaling pathways. In fact, a previous study reported that compared to wt mice, PTX3 ko mice showed a decrease in VEGF receptor 2 (VEGFR2),^53^ which might cause decreased angiogenesis in the ko mice. However, our preliminary data showed that 1 week of PTX3 suppression by siRNA did not change the expression level of VEGFR2 in cerebral white matter (data not shown). Therefore, to pursue the therapeutic potential of PTX3 signaling, we need to carefully dissect the mechanisms by which PTX3 regulates the cerebrovascular system after CNS diseases.

In summary, this study demonstrates that PTX3 is an important NVU mediator for stroke pathology in white matter by showing the biphasic roles of PTX3 after white matter stroke—beneficial during the acute phase, but detrimental during the chronic phase. Our current findings support the idea that PTX3 would be an attractive therapeutic target for white matter‐related disease, including stroke. Additionally, a deeper understanding of PTX3 roles would help to elucidate the complex mechanisms of stroke pathology.

## CONFLICT OF INTEREST

None.

## Supporting information

App S1

## Data Availability

The data that support the findings of this study are available from the corresponding author upon reasonable request.

## References

[1] Ronaldson PT , Davis TP . Regulation of blood‐brain barrier integrity by microglia in health and disease: a therapeutic opportunity. J Cereb Blood Flow Metab. 2020;40(1_suppl):S6‐S24. 32928017 10.1177/0271678X20951995 PMC7687032

[2] Moskowitz MA , Lo EH , Iadecola C . The science of stroke: mechanisms in search of treatments. Neuron. 2010;67(2):181‐198. 20670828 10.1016/j.neuron.2010.07.002 PMC2957363

[3] Zhu Z , Zheng L , Li Y , et al. Potential immunotherapeutic targets on myeloid cells for neurovascular repair after ischemic stroke. Front Neurosci. 2019;13:758. 31447626 10.3389/fnins.2019.00758 PMC6696904

[4] Ohtomo R , Arai K . Recent updates on mechanisms of cell‐cell interaction in oligodendrocyte regeneration after white matter injury. Neurosci Lett. 2020;715:134650. 31770564 10.1016/j.neulet.2019.134650 PMC6936598

[5] Terasaki Y , Liu Y , Hayakawa K , et al. Mechanisms of neurovascular dysfunction in acute ischemic brain. Curr Med Chem. 2014;21(18):2035‐2042. 24372202 10.2174/0929867321666131228223400 PMC4066327

[6] Banks WA , Kovac A , Morofuji Y . Neurovascular unit crosstalk: pericytes and astrocytes modify cytokine secretion patterns of brain endothelial cells. J Cereb Blood Flow Metab. 2018;38(6):1104‐1118. 29106322 10.1177/0271678X17740793 PMC5998993

[7] Esposito E , Li W , Xing C , Lo EH . Help‐me signaling as a paradigm for inter‐cellular effects of pre‐ and post‐conditioning in the brain after stroke. Conditioning Med. 2018;1(7):337‐342.

[8] Lo EH . Degeneration and repair in central nervous system disease. Nat Med. 2010;16(11):1205‐1209. 21052074 10.1038/nm.2226 PMC3985732

[9] Lo EH . A new penumbra: transitioning from injury into repair after stroke. Nat Med. 2008;14(5):497‐500. 18463660 10.1038/nm1735

[10] Zhang ZG , Chopp M . Neurorestorative therapies for stroke: underlying mechanisms and translation to the clinic. Lancet Neurol. 2009;8(5):491‐500. 19375666 10.1016/S1474-4422(09)70061-4 PMC2727708

[11] Magaki SD , Williams CK , Vinters HV . Glial function (and dysfunction) in the normal & ischemic brain. Neuropharmacology. 2018;134(Pt B):218‐225. 29122627 10.1016/j.neuropharm.2017.11.009 PMC6132239

[12] Zhang ZG , Zhang L , Jiang Q , et al. VEGF enhances angiogenesis and promotes blood‐brain barrier leakage in the ischemic brain. J Clin Invest. 2000;106(7):829‐838. 11018070 10.1172/JCI9369 PMC517814

[13] Navaratna D , Guo S , Arai K , Lo EH . Mechanisms and targets for angiogenic therapy after stroke. Cell Adh Migr. 2009;3(2):216‐223. 19363301 10.4161/cam.3.2.8396 PMC2679890

[14] Seo JH , Miyamoto N , Hayakawa K , et al. Oligodendrocyte precursors induce early blood‐brain barrier opening after white matter injury. J Clin Investig. 2013;123(2):782‐786. 23281396 10.1172/JCI65863 PMC3561802

[15] Maki T , Hayakawa K , Pham LD , Xing C , Lo EH , Arai K . Biphasic mechanisms of neurovascular unit injury and protection in CNS diseases. CNS Neurol Disord Drug Targets. 2013;12(3):302‐315. 23469847 10.2174/1871527311312030004 PMC3845030

[16] Zhao BQ , Wang S , Kim HY , et al. Role of matrix metalloproteinases in delayed cortical responses after stroke. Nat Med. 2006;12(4):441‐445. 16565723 10.1038/nm1387

[17] Chan SJ , Esposito E , Hayakawa K , et al. vascular endothelial growth factor 165‐binding heparan sulfate promotes functional recovery from cerebral ischemia. Stroke. 2020;51(9):2844‐2853. 32772683 10.1161/STROKEAHA.119.025304

[18] Haupt M , Zechmeister B , Bosche B , et al. Lithium enhances post‐stroke blood‐brain barrier integrity, activates the MAPK/ERK1/2 pathway and alters immune cell migration in mice. Neuropharmacology. 2020;181:108357. 33065166 10.1016/j.neuropharm.2020.108357

[19] Shindo A , Maki T , Mandeville ET , et al. Astrocyte‐derived pentraxin 3 supports blood‐brain barrier integrity under acute phase of stroke. Stroke. 2016;47(4):1094‐1100. 26965847 10.1161/STROKEAHA.115.012133 PMC4811738

[20] Bonsack B , Borlongan MC , Lo EH , Arai K . Brief overview: protective roles of astrocyte‐derived pentraxin‐3 in blood‐brain barrier integrity. Brain Circ. 2019;5(3):145‐149. 31620663 10.4103/bc.bc_37_19 PMC6785941

[21] Rajkovic I , Denes A , Allan SM , Pinteaux E . Emerging roles of the acute phase protein pentraxin‐3 during central nervous system disorders. J Neuroimmunol. 2016;292:27‐33. 26943955 10.1016/j.jneuroim.2015.12.007

[22] Ryu WS , Kim CK , Kim BJ , Kim C , Lee SH , Yoon BW . Pentraxin 3: a novel and independent prognostic marker in ischemic stroke. Atherosclerosis. 2012;220(2):581‐586. 22178425 10.1016/j.atherosclerosis.2011.11.036

[23] Qin LZ , Li W , Huang Y , et al. PTX3 expression in the plasma of elderly ACI patients and its relationship with severity and prognosis of the disease. Eur Rev Med Pharmacol Sci. 2016;20(19):4112‐4118. 27775787

[24] Sezer S , Ucar F , Ulusoy EK , et al. Serum amyloid A, fetuin‐A, and pentraxin‐3 levels in patients with ischemic stroke: novel prognostic biomarkers? Turk J Med Sci. 2014;44(1):16‐23. 25558553 10.3906/sag-1211-90

[25] Shindo A , Tanemura H , Yata K , et al. Inflammatory biomarkers in atherosclerosis: pentraxin 3 can become a novel marker of plaque vulnerability. PLoS One. 2014;9(6):e100045. 24936646 10.1371/journal.pone.0100045 PMC4061039

[26] Benowitz LI , Carmichael ST . Promoting axonal rewiring to improve outcome after stroke. Neurobiol Dis. 2010;37(2):259‐266. 19931616 10.1016/j.nbd.2009.11.009 PMC2818530

[27] Jiang Q , Zhang ZG , Chopp M . MRI evaluation of white matter recovery after brain injury. Stroke. 2010;41(10 Suppl):S112‐S113. 20876482 10.1161/STROKEAHA.110.595629 PMC2953715

[28] Yang Y , Rosenberg GA . Blood‐brain barrier breakdown in acute and chronic cerebrovascular disease. Stroke. 2011;42(11):3323‐3328. 21940972 10.1161/STROKEAHA.110.608257 PMC3584169

[29] Tosto G , Zimmerman ME , Carmichael OT , Brickman AM . Predicting aggressive decline in mild cognitive impairment: the importance of white matter hyperintensities. JAMA Neurol. 2014;71(7):872. 24821476 10.1001/jamaneurol.2014.667 PMC4107926

[30] Philips T , Rothstein JD . Glial cells in amyotrophic lateral sclerosis. Exp Neurol. 2014;262:111‐120. 24859452 10.1016/j.expneurol.2014.05.015 PMC4241182

[31] Rost NS , Cougo P , Lorenzano S , et al. Diffuse microvascular dysfunction and loss of white matter integrity predict poor outcomes in patients with acute ischemic stroke. J Cereb Blood Flow Metab. 2018;38(1):75‐86. 10.1177/0271678X17706449 28481164 PMC5757442

[32] Corbin ZA , Rost NS , Lorenzano S , et al. White matter hyperintensity volume correlates with matrix metalloproteinase‐2 in acute ischemic stroke. J Stroke Cerebrovasc Dis. 2014;23(6):1300‐1306. 24439130 10.1016/j.jstrokecerebrovasdis.2013.11.002 PMC4058345

[33] Arai K . Can oligodendrocyte precursor cells be a therapeutic target for mitigating cognitive decline in cerebrovascular disease? J Cereb Blood Flow Metab. 2020;40(8):1735‐1736. 32674700 10.1177/0271678X20929432 PMC7370360

[34] Rosenzweig S , Carmichael ST . Age‐dependent exacerbation of white matter stroke outcomes: a role for oxidative damage and inflammatory mediators. Stroke. 2013;44(9):2579‐2586. 23868277 10.1161/STROKEAHA.113.001796 PMC3791618

[35] Blasi F , Wei Y , Balkaya M , et al. Recognition memory impairments after subcortical white matter stroke in mice. Stroke. 2014;45(5):1468‐1473. 24723319 10.1161/STROKEAHA.114.005324

[36] Maki T , Morancho A , Martinez‐San Segundo P , et al. Endothelial progenitor cell secretome and oligovascular repair in a mouse model of prolonged cerebral hypoperfusion. Stroke. 2018;49(4):1003‐1010. 29511131 10.1161/STROKEAHA.117.019346 PMC5871569

[37] Koh SH , Liang AC , Takahashi Y , et al. Differential effects of isoxazole‐9 on neural stem/progenitor cells, oligodendrocyte precursor cells, and endothelial progenitor cells. PLoS One. 2015;10(9):e0138724. 26407349 10.1371/journal.pone.0138724 PMC4583469

[38] Arai K , Jin G , Navaratna D , Lo EH . Brain angiogenesis in developmental and pathological processes: neurovascular injury and angiogenic recovery after stroke. FEBS J. 2009;276(17):4644‐4652. 19664070 10.1111/j.1742-4658.2009.07176.x PMC3712842

[39] Pham LD , Hayakawa K , Seo JH , et al. Crosstalk between oligodendrocytes and cerebral endothelium contributes to vascular remodeling after white matter injury. Glia. 2012;60(6):875‐881. 22392631 10.1002/glia.22320 PMC3325331

[40] Rosell A , Lo EH . Multiphasic roles for matrix metalloproteinases after stroke. Curr Opin Pharmacol. 2008;8(1):82‐89. 18226583 10.1016/j.coph.2007.12.001

[41] Greenberg DA . Neurogenesis and stroke. CNS Neurol Disord: Drug Targets. 2007;6(5):321‐325. 18045160 10.2174/187152707783220901

[42] Argaw AT , Asp L , Zhang J , et al. Astrocyte‐derived VEGF‐A drives blood‐brain barrier disruption in CNS inflammatory disease. J Clin Invest. 2012;122(7):2454‐2468. 22653056 10.1172/JCI60842 PMC3386814

[43] Ronca R , Giacomini A , Di Salle E , et al. Long‐Pentraxin 3 derivative as a small‐molecule FGF trap for cancer therapy. Cancer Cell. 2015;28(2):225‐239. 26267536 10.1016/j.ccell.2015.07.002

[44] Zheng Z , Chopp M , Chen J . Multifaceted roles of pericytes in central nervous system homeostasis and disease. J Cereb Blood Flow Metab. 2020;40(7):1381‐1401. 32208803 10.1177/0271678X20911331 PMC7308511

[45] Sencan I , Esipova T , Kilic K , et al. Optical measurement of microvascular oxygenation and blood flow responses in awake mouse cortex during functional activation. J Cereb Blood Flow Metab. 2020 10.1177/0271678X20928011. [Epub ahead of print]. PMC8985437 32515672

[46] Rust R . Insights into the dual role of angiogenesis following stroke. J Cereb Blood Flow Metab. 2020;40(6):1167‐1171. 32065073 10.1177/0271678X20906815 PMC7238380

[47] Kanazawa M , Takahashi T , Ishikawa M , Onodera O , Shimohata T , Del Zoppo GJ . Angiogenesis in the ischemic core: a potential treatment target? J Cereb Blood Flow Metab. 2019;39(5):753‐769. 30841779 10.1177/0271678X19834158 PMC6501515

[48] Balkaya M , Cho S . Optimizing functional outcome endpoints for stroke recovery studies. J Cereb Blood Flow Metab. 2019;39(12):2323‐2342. 31522590 10.1177/0271678X19875212 PMC6893977

[49] Zhang ZG , Chopp M . Promoting brain remodeling to aid in stroke recovery. Trends Mol Med. 2015;21(9):543‐548. 26278490 10.1016/j.molmed.2015.07.005 PMC4567429

[50] Napoli E , Lippert T , Borlongan CV . Stem cell therapy: repurposing cell‐based regenerative medicine beyond cell replacement. Adv Exp Med Biol. 2018;1079:87‐91. 29480446 10.1007/5584_2018_174

[51] Lee J , Hamanaka G , Lo EH , Arai K . Heterogeneity of microglia and their differential roles in white matter pathology. CNS Neurosci Ther. 2019;25(12):1290‐1298. 31733036 10.1111/cns.13266 PMC6887901

[52] Kang M , Yao Y . Oligodendrocytes in intracerebral hemorrhage. CNS Neurosci Ther. 2019;25(10):1075‐1084. 31410988 10.1111/cns.13193 PMC6776757

[53] Rodriguez‐Grande B , Varghese L , Molina‐Holgado F , et al. Pentraxin 3 mediates neurogenesis and angiogenesis after cerebral ischaemia. J Neuroinflammation. 2015;12:15. 25616391 10.1186/s12974-014-0227-y PMC4308938

