# Supplementary material for: Biphasic roles of pentraxin 3 in cerebrovascular function after white matter stroke
Source: CNS Neurosci Ther. 2020 Dec 11;27(1):60–70. doi: 10.1111/cns.13510 (PMC7804900; doi:10.1111/cns.13510)
Supplement: Supplementary file 1 — App S1 [file CNS-27-60-s001.doc]

**Appendix S1**

**Supplementary Information**

**Biphasic roles of pentraxin-3 in cerebrovascular function after white matter stroke**

Akihiro Shindo^1,2^, Hajime Takase^1^, Gen Hamanaka^1^, Kelly K. Chung^1^ Emiri T. Mandeville^1^, Naohiro Egawa^1,3^, Takakuni Maki^1,3^, Mia Borlongan^1^, Ryosuke Takahashi^3^, Josephine Lok^1,4^, Hidekazu Tomimoto^2^, Eng H. Lo^1^, Ken Arai^1^

^1^ Neuroprotection Research Laboratory, Departments of Radiology and Neurology, Massachusetts General Hospital and Harvard Medical School, Charlestown, Massachusetts, USA

^2^ Department of Neurology, Mie University Graduate School of Medicine, Japan

^3^ Department of Neurology, Graduate School of Medicine, Kyoto University, Japan

^4^ Pediatric Critical Care Medicine, Massachusetts General Hospital, Boston, MA, USA

Corresponding author: Ken Arai, Neuroprotection Research Laboratory, MGH East 149-2401, Charlestown, MA 02129, USA. Tel: 617.724.9503, Email: karai@mgh.harvard.edu

Content:

- Supplementary Figures
- Un-clopped western blot images

**Supplementary Figure S1. Efficacy of PTX3 siRNA in mice:** Male mice were treated with control siRNA or PTX3 siRNA. Western blotting confirmed that PTX3 siRNA downregulated PTX3 expression in the affected region at least for 5 days. Data are mean + SD from N=4. *p < 0.05 (One-way ANOVA followed by post-hoc Dunnett’s multiple comparison test).


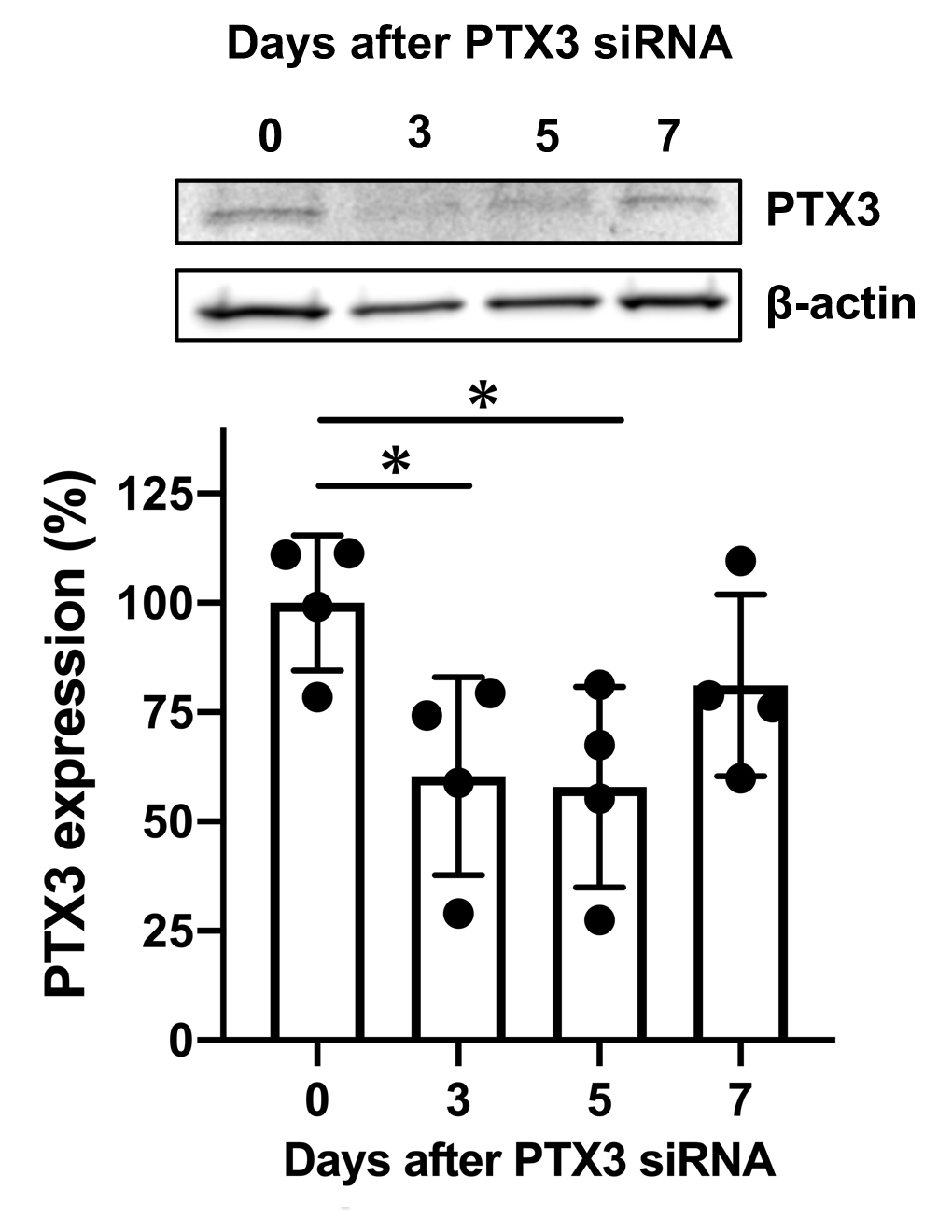


**Supplementary Figure S2:** Double staining of ZO-1 with CD31 showed that ZO-1 expression was observed in CD31-positive cerebral vessels in mouse corpus callosum.

**Supplementary Figure S3. Experimental schedule to examine the roles of PTX3 in compensatory angiogenesis in mice:** Four experimental groups were prepared. Mice in the sham group received the sham operation (PBS injection into corpus callosum, instead of ET-1 injection) at day 0, and then at day 7, they were treated with control siRNA. Mice in the control group received ET-1 injection at day 0, and then at day 7, they were treated with control siRNA. Mice in the PTX3-siRNA (@day7) group received ET-1 injection at day 0, and then at day 7, they were treated with control siRNA. Finally, as a negative control, we prepared one more group (PTX3-siRNA (@day0) group) that received ET-1 injection after the treatment of PTX3 siRNA at day 0.

**Supplementary Figure S4. Biphasic roles of PTX3 in a mouse model of white matter stroke by L-Nio injection: (a-d)** Male mice were subjected to L-Nio injection after the treatment of control-siRNA or PTX3-siRNA. Three days after L-Nio injection, white matter stroke mice were sacrificed, and brain samples were used for immunostaining. Mice that received PTX3 siRNA exhibited less myelin density and more BBB damage, compared to mice with control siRNA at day 3 after L-Nio injection. Scale bar = 100 μm for (a) or 50 μm for (b). Data are mean + SD from N=8. *p < 0.05. **(c)** Male mice were subjected to L-Nio injection, and 7 days later, they were treated with control-siRNA or PTX3-siRNA. Twenty-one days after L-Nio injection, white matter stroke mice were sacrificed, and brain samples were used for immunostaining. Mice that received PTX3 siRNA exhibited more signals of Collagen-IV (endothelial marker) at day 21 after L-Nio injection. Scale bar = 100 μm. Data are mean + SD from N=8. *p < 0.05 (Unpaired t test with Welch’s correction for (a) and (c), Mann Whitney test for (b)).


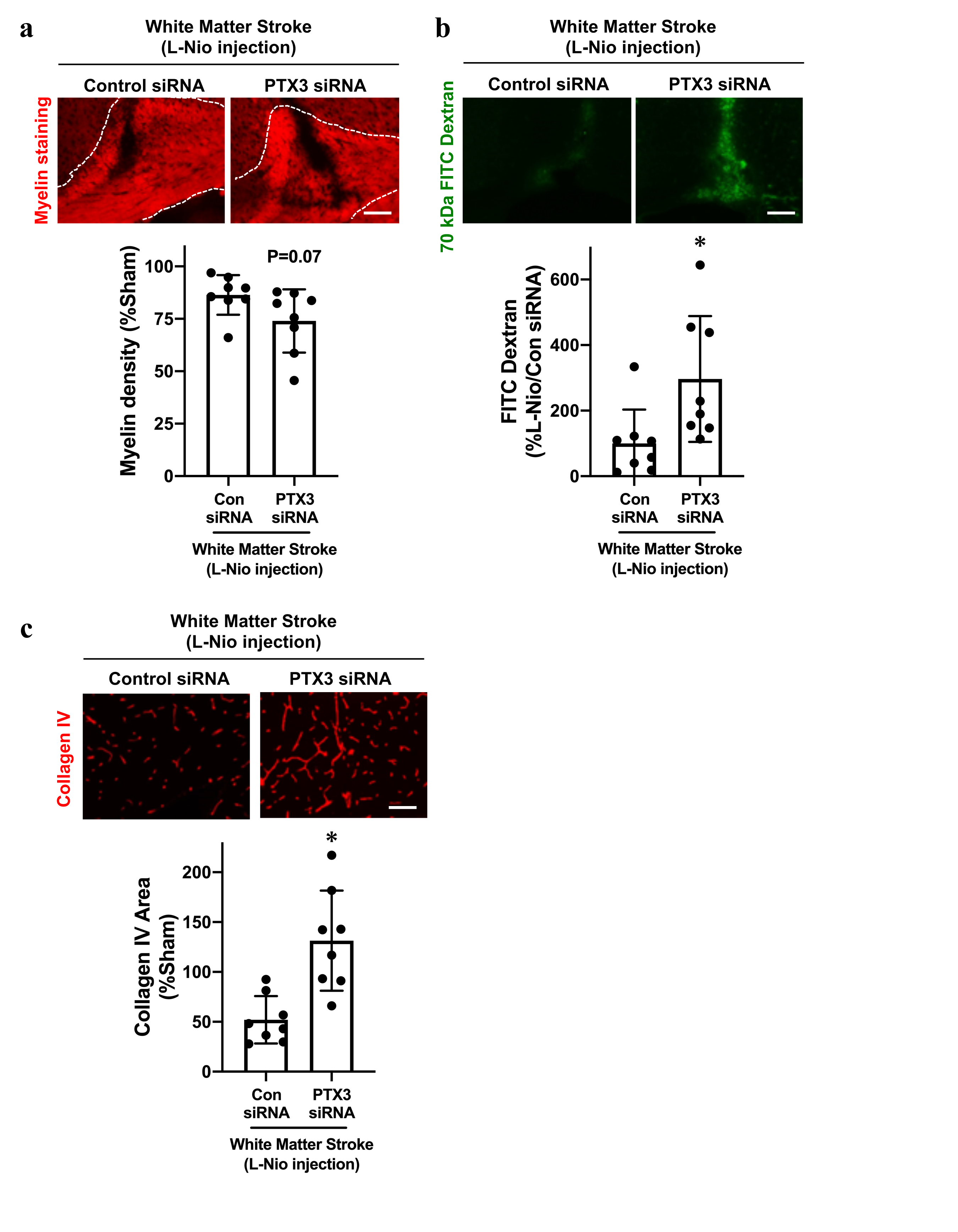


**Un-cropped western blot images for Figure 1d:**

**Un-cropped western blot images for Figure 2a:**

**Un-cropped western blot images for Figure 4a:**

**Un-cropped western blot images for Supplementary Figure S1a:**
